# Calcineurin inhibitors’ impact on cardiovascular and renal function, a descriptive study in lung transplant recipients from the North of Spain

**DOI:** 10.1038/s41598-022-25445-2

**Published:** 2022-12-08

**Authors:** Rita Nogueiras-Álvarez, Víctor Manuel Mora-Cuesta, José Manuel Cifrián-Martínez, María Ángeles de Cos-Cossío, María del Mar García-Sáiz

**Affiliations:** 1 MD. Clinical pharmacologist. PhD candidate, Cantabria University, Santander, Spain; 2 Pneumology Service. Lung Transplant Unit, Marqués de Valdecilla University Hospital, 39008 Santander, Spain; 3 Clinical Pharmacology Service, Marqués de Valdecilla University Hospital, 39008 Santander, Spain

**Keywords:** Medical research, Risk factors, Adverse effects, Drug therapy, Health care, Therapeutics, Comorbidities

## Abstract

Patients undergoing lung transplantation (LTx) need administration of immunosuppressive therapy following the procedure to prevent graft rejection. However, these drugs are not exempt from potential risks. The development of cardiovascular risk factors and impaired renal function in the post-transplantation period are conditions that may be favoured by the use of calcineurin inhibitor (CNI) drugs which could have repercussions on the quality of life and the post-transplantation evolution. To evaluate the cardiovascular and renal toxicity following the administration of CNI as maintenance immunosuppression in lung transplant recipients (LTRs) we reviewed a total number of 165 patients undergoing LTx between 01/01/2015 and 08/12/2018. They were divided into two groups according to the CNI drug administrated: cyclosporine (CsA-group) with 11 patients or tacrolimus (Tac-group), with 154 patients. We evaluated the de novo occurrence of arterial hypertension (HTN), diabetes mellitus (DM), hyperlipidemia and impaired renal function after initiation of CNI administration. In addition to that, the time until each of these events was assessed. A higher rate for developing HTN (*p* < 0.001) and impaired renal function (*p* = 0.047) was observed within the CsA-group. The new onset of hyperlipidemia was similar between both CNI groups and de novo appearance of DM was only documented in those LTRs receiving tacrolimus. In this LTRs retrospective study, it was observed that having ≥ 4 tacrolimus trough levels above the upper limit of the proposed interval for each specific post-LTx period was associated with an increased risk for developing renal impairment. No other statistically significant association was found between supratherapeutic CNIs blood levels and the evaluated toxicities.

## Introduction

Patients with end-stage lung disease who undergo lung transplantation (LTx) are expected to improve their life expectancy and quality of life^1^ after the procedure.

According to the International Society for Heart and Lung Transplantation (ISHLT) Registry, in recent years the most used immunosuppressive regimen in lung transplant recipients (LTRs) includes prednisone, mycophenolate mofetil (or mycophenolic acid) and tacrolimus. Although cyclosporine is still used in certain recipients, there has been a progressive decline in its administration compared to tacrolimus in recent times^2^ because tacrolimus has demonstrated increased lung graft and recipient survival^3^.

It is necessary to be aware of the toxicities that may result from the use of these drugs in the post-transplantation management of these patients in order to cope with them.

In the case of LTx, a number of specific assumptions must also be considered: this regimen must be maintained for life and the intensity of immunosuppressive therapy is usually stronger due to the higher risk of rejection compared to other types of solid organ transplantation (SOT)^4^.

Because of the above mentioned issues, LTRs are exposed to a higher risk of toxicities from immunosuppressive therapy.

### Toxicities related with the use of CNIs in LTx

Although the information of immunosuppression effects is usually mostly derived from results observed in other types of transplantation^5^, in LTRs with long-term survival, particular attention should be focused on the cardiovascular toxicities such as arterial hypertension (HTN), hyperlipidemia and diabetes mellitus (DM)_ and also to the development of impaired renal function. These comorbidities should be taken into account as they relate with increased morbidity and decreased survival after transplantation^6^.

Despite its importance, the incidence of these comorbidities is no longer reported in the annual reports published by ISHLT in the area of LTx. In fact, the 2016 annual report^7^ was the last one where information on the development of post-LTx HTN, renal dysfunction, hyperlipidemia and DM was collected. The 2017, 2018 and 2019 annual reports only contained data on renal dysfunction and DM, but there is no longer any reference to the development of those comorbidities in the following annual reports.

#### CNIs-related cardiovascular toxicity

The risk of developing HTN, hyperlipidemia and/or DM increases in post-transplant recipients in direct association with the use of immunosuppressive drugs^6^.

When evaluating the percentage of deaths due to cardiovascular causes in LTRs after the first month post-transplantation, the percentage is estimated to be around 5–6%^2^.

However, it should be noted that this low percentage may be related to the smaller incidence of cardiovascular events in LTRs in relation to their lower survival rate when compared to other SOT. In this regard, assessing overall survival after transplantation, the ISHLT reports that for adults who had undergone primary LTx in recent years (data obtained between 2010-June 2017), the median survival is 6.7 years^2^; while the median survival time among kidney recipients has been reported to be 12.4 years; 11.1 years in the case of adult cadaveric liver transplantation and 9.4 years for adult heart transplantation^8^.

##### Arterial hypertension

CNIs are known to raise blood pressure through different mechanisms: vasoconstriction of the afferent renal arteriole (increasing sodium and water reabsorption at the level of the renal tubule, that leads to volume expansion and increases blood pressure)^9^, mechanisms related to the sympathetic nervous system^10^, alterations in regulation of intracellular calcium ions, excess production of vasoconstrictors and reduction in the production of vasodilatory prostaglandins^7^.

In addition to the risk associated with the use of CNI, in transplant recipients there are other immunosuppressive drugs that have also proven to increase blood pressure, such as corticosteroids (due to their mineralocorticoid effect and their contribution to increase vascular resistance and cardiac contractility)^11^ or mammalian target of rapamycin (mTOR) inhibitors when combined with CNIs^12^.

##### Hyperlipidemia

The occurrence of hyperlipidemia is a common condition after transplantation. But it is important to bear in mind that in addition to CNIs, other drugs used in the post-transplantation immunosuppressive protocol may contribute to the development of hyperlipidemia, such as corticosteroids or mTOR inhibitors^13^.

It has been reported that the increase in total cholesterol concentration may be of particular significance in the earliest post-transplant stage, especially between 3 and 6 months after transplantation^14^, although significant increases may occur throughout the first-year post-transplantation.

The ISHLT registry reported de novo development of this comorbidity for the last time in 2016, where it was stated that up to 20–30% LTRs developed hyperlipidemia beyond the first year post-LTx in most cases^6^. This percentage is lower than that documented in other types of SOT. As an example, for cardiac transplant recipients who survive the first year, hyperlipidemia has been reported to be 60% and this rate was 88% for those who survived beyond 5 years^15^.

##### Diabetes mellitus

The development of de novo DM in the post-transplant period is sometimes referred to by the term NODAT, which stands for new onset diabetes mellitus after transplant.

The mechanisms by which CNIs produce this alteration in glucose metabolism are multiple. It has been documented that these drugs produce an alteration in pancreatic beta cells that lead to a reduction in insulin secretion, and it has also been observed that there is evidence of an increased resistance to the effect of insulin in the rest of the body's cells^16^. In relation to impaired insulin secretion, this effect may in turn be related to another common side effect of CNIs themselves such as hypomagnesemia, which contributes to this impairment^17^ by promoting increased calcium levels in pancreatic beta cells^18^.

Although, as mentioned, CNIs are drugs that favor the development of DM, it is important to note that they are not the only ones to be considered when talking about transplant recipients. Corticosteroids, commonly used as part of post-transplant immunosuppressive maintenance treatment, are also known to favor the development of NODAT when used over a prolonged period of time^19^. Moreover, it should not be forgotten that the use of methylprednisolone boluses is a common practice in the treatment of acute rejection episodes, which significantly increases the dose of corticosteroid exposure in recipients who suffer rejection^20^.

#### CNIs-related nephrotoxicity

Although there are other causes in the early post-transplant period that may lead to the development of renal function impairment that should not be overlooked (such as hemodynamic instability, inflammation and the administration of non-steroidal anti-inflammatory drugs as painkillers), it is important to consider renal impairment caused by CNIs as well.

CNIs nephrotoxicity is related to vasoconstriction of afferent and efferent arterioles^21^ and an inadequate vasodilatory response, particularly in the afferent arteriole^22^. When CNIs concentration in whole blood is elevated, this vasoconstriction is thought to be potentiated, leading to acute kidney damage^23^. In the specific field of LTx, supratherapeutic tacrolimus levels have been considered to be related to the development of renal failure^24,25^. However, it should not be forgotten that nephrotoxicity can occur even with immunosuppressant blood levels within the therapeutic range, when there is a high plasma fraction of CNI that is not bound to proteins^26–28^.

The magnitude of this issue is noteworthy, and it is widely recognized that chronic kidney disease among non-renal SOTs often requires renal replacement techniques such as dialysis or even kidney transplantation^29^. When reviewing prevalence of chronic kidney disease among LTRs, values range from 15 to 20% after 5 years and kidney transplantation has also been pointed a reasonable option^30^.

### CNIs blood levels and post-transplant evolution

In the post-transplantation period, CNIs therapeutic drug monitoring is essential to assess recipients' level of immunosuppression.

There are reference intervals to be followed depending on the type of transplant and the post-transplant stage of the recipient.

It is important to attempt that the immunosuppression blood level is the appropriate for each post-transplantation stage, avoiding both levels that are below the reference interval (which are related to risk of rejection) and levels that are above the reference interval (which are related to an increased risk of infections, malignancy or with the development of toxicities^31–33^.

The optimal immunosuppression blood level is difficult to achieve following transplantation and this fact may partially explain the frequent failure of the immunosuppressive strategy after LTx (50–60% of the recipients develop acute rejection and up to 60% of LTRs who survive more than 5 years are affected by bronchiolitis obliterans syndrome)^34,35^.

### Objectives

The aim of this study was to evaluate different toxicities following the administration of CNI drugs as maintenance immunosuppression in LTx and to assess whether blood levels of immunosuppressants are related to the occurrence of these events.

## Patients and methods

### Study population

We examined recipients who received their lung allografts at the Marqués de Valdecilla University Hospital (HUMV) between 1 January 2015 and 8 December 2018.

We conducted a retrospective study using the electronic medical records of the recipients and the LTx database of the center as sources of information.

Inclusion criteria were as follows: (1) having had LTx; (2) being aged 18 years or above at the moment of LTx; (3) post-transplant immunosuppressive treatment regimen that included a CNI drug.

Patients presenting any of the following criteria were not selected for participation in this study: (1) patients who were not prescribed immunosuppressive treatment with CNIs; (2) patients who had previously expressed their refusal to participate in research studies.

As demographic variables, we included data on the gender of the recipients, their age at the time of transplantation and specific transplant-related data: type of LTx and underlying lung disease.

We evaluated the de novo occurrence of HTN, hyperlipidemia, DM and impaired renal function after initiation of immunosuppressive therapy with CNIs. In addition, we assessed the time until each of these events.

### Immunosuppression protocol for LTx and CNIs blood level determination in our center

In our center, the standard immunosuppression protocol consists of a triple immunosuppressive therapy that includes a CNI (preferably tacrolimus), mycophenolate mofetil or mycophenolic acid and corticosteroids. In April 2016 basiliximab induction started to be used routinely. Before that date, it was just employed in selected cases, such as elderly patients or in cases of renal dysfunction or severe pulmonary hypertension.

The blood level determinations of CNI in our center are routinely performed in the Clinical Pharmacology laboratory by chemiluminescent microparticle immunoassay in an ARCHITECT i-1000^®^ platform (Abbot Diagnostics).

### Definitions: de novo toxicities

In this study, we classified as LTRs with appearance of de novo HTN those patients who were prescribed an antihypertensive drug due to confirmation of sustained elevated arterial blood pressure levels.

Recipients were classified with de novo development of hyperlipidemia if they were prescribed a statin due to confirmation of elevated cholesterol levels, the upper limit of the reference range being set at 200 mg/dL in our center laboratory.

We defined de novo onset DM when elevated post-transplant blood glucose levels were documented and required some sort of corrective intervention (including pharmacological treatment with insulin or oral hypoglycemic agents).

The category of recipient with impaired renal function was given to those patients with a marked increase in serum creatinine and a significant decrease in glomerular filtration rate compared to their pre-transplant baseline (any decrease in renal function status in KDIGO classification present for > 3 months). The upper limit of the reference range for the serum creatinine value in the laboratory of our center is 1.18 mg/dL. The glomerular filtration rate is calculated using the CKD-EPI (Chronic Kidney Disease Epidemiology Collaboration) formula, where ≥ 90 mL/min/1.73 m^2^ is assumed to be normal.

The date of onset of these events was defined as the date on which the event was first recorded in the clinical history, either as a diagnosis or as first reference made to the administration of a specific treatment.

### Relationship between CNI blood levels and the development of comorbidities

We evaluated CNI blood levels at specific timepoints on dates coinciding with routine post-LTx control examinations: 3rd month, 6th month, 9th month, 1st year, 18 months, 2nd year and 3rd year. Thus, the maximum number of analytical results available per patient was seven.

These are the points that were selected for this study although the follow-up of LTRs includes many more evaluation points, which are particularly frequent in the immediate post-transplantation period. In our center these evaluations are set every 2 weeks for the first 6 months; every month in the period from 6 to 12 months; once every 2 months between 12 and 24 months and once every 3 months beyond 24 months, as well as whenever there is a clinical deterioration.

For each blood level test, we assessed whether the result obtained was within or above the therapeutic range proposed for the specific post-LTx stage.

For this purpose, each specific result was assessed on a case-by-case basis, considering the CNI drug that the patient was receiving.

For tacrolimus it is usually determined the trough level or C0, which is the level just before the dose is taken. In LTx, the trough levels of tacrolimus are maintained between 10 and 15 mcg/L the first 12 months and afterwards the reference interval is established at 8–10 mcg/L.

For cyclosporine, blood determinations can be made both at the trough level (C0) and 2 h after the dose is taken (C2). The reference blood levels also differ according to the post-LTx stage: first 6 months after the procedure (C0: 250–350 mcg/L and C2: 800–1000 mcg/L), 6–12 months post-LTx (C0: 200–250 mcg/L and C2: 600–800 mcg/L) or > 12 months (C0: 125–250 mcg/L and C2: 400–600 mcg/L).

### Data/statistical analysis

For the data analysis Software IBM SPSS Statistics 20 was used.

Qualitative variables were expressed as frequencies and percentages, while quantitative variables, depending on their homogeneity, were expressed as the mean ± standard deviation (in the case of those which followed a normal distribution) or median with the interquartile range (in the case of those with a non-normal distribution). To determine if the continuous quantitative variables followed a normal distribution the Smirnov–Kolmogorov test or the Shapiro–Wilk test were used.

To compare quantitative variables, the Student's t-test and the Mann–Whitney U-test were used, depending on the homogeneity of the parameters.

For categorical variables, chi-square or Fisher's exact test were used.

Analysis on CNI blood levels and their association with each of the studied events was performed and the results are given as an odds ratio (OR). The confidence interval (CI) was established at 95%.

Finally, a log-rank test was carried out to study each of the different event-free periods (onset of HTN, hyperlipidemia, DM and impaired renal function). These findings were represented with Kaplan–Meier graphs. For time-to-event analysis, follow-up of recipients not experiencing the event of interest were censored.

A probability value of less than 0.05 was considered statistically significant.

### Institutional review board statement

The study was conducted in accordance with the Declaration of Helsinki, and approved by the Drug Research Ethics Committee of Cantabria (Spain), with protocol code IDI-ICN-2020-01 (date of approval: 07/september/2020).

### Informed consent

Patient consent was waived due to the retrospective, observational nature of this study.

## Results

We reviewed a total number of 165 LTRs. From the total group of patients reviewed, 109 men and 56 women were included. The additional demographic characteristics are shown in Table 1.

**Table 1:** LTRs demographic features.

Characteristics	n = 165	Cyclosporine treated LTRs (n = 11)	Tacrolimus treated LTRs (n = 154)	p*
**Age at the time of LTx** (years)	58.09 (50.08–62.28)	58.09 (52.24–60.86)	58.25 (49.71–62.32)	0.855
**Gender**				
Male	109 (66.10%)	6 (54.50%)	103 (66.90%)	0.512
Female	56 (33.90%)	5 (45.50%)	51 (33.10%)	
**Type of lung transplantation**				
Single lung transplant	54 (32.70%)	3 (27.30%)	51 (33.10%)	1.000
Double lung transplant	111 (67.30%)	8 (72.70%	103 (66.90%)	
**Underlying lung disease**				
Chronic obstructive pulmonary disease	53 (32.10%)	3 (27.30%)	50 (32.50%)	**0.006**
Interstitial lung disease	84 (50.90%)	7 (63.60%)	77 (50.00%)	
Bronchiectasis/cystic fibrosis	17 (10.30%)	–	17 (11.00%)	
Pulmonary arterial hypertension	6 (3.60%)	1 (9.10%)	5 (3.20%)	
Re-transplantation	2 (1.20%)	–	2 (1.30%)	
Others	3 (1.80%)	–	3 (1.00%)	
**Pre-transplant comorbidities**				
HTN	32 (19.40%)	–	32 (20.80%)	0.125
Hyperlipidemia	38 (23.00%)	1 (9.09%)	37 (24.00%)	0.460
DM	17 (10.30%)	–	17 (11.04%)	
Type 1 DM	3 (1.80%)	–	3 (1.95%)	1.000
Type 2 DM	14 (8.50%)	–	14 (9.09%)	0.601
Impaired renal function	2 (1.20%)	–	2 (1.30%)	1.000
**Basiliximab induction**				
Yes	119 (72.10%)	5 (45.50%)	114 (74.00%)	0.074
No	46 (27.90%)	6 (54.50%)	40 (26.00%)	

*HTN* arterial hypertension, *DM* diabetes mellitus, *LTRs* lung transplant recipients, *LTx* lung transplantation.

*The boldface type indicates differences that are statistically significant (*p* < 0.05).

Patients were divided into 2 groups according to the CNI drug: cyclosporine (CsA-group) or tacrolimus (Tac-group). The study included 11 patients treated with cyclosporine (6.67%) and 154 patients treated with tacrolimus (93.33%).

Among the 11 LTRs treated with cyclosporine, all but one of them (due to a previous diagnosis of allergy to macrolides and tacrolimus), were initially on an immunosuppressive regimen with tacrolimus since in our center, according to our protocol, tacrolimus is used as the CNI of choice in first instance, and the switch to cyclosporine is only made in case of very specific situations. The reason for the change from tacrolimus to cyclosporine in these 10 LTRs was the suspicion of neurotoxicity in relation to tacrolimus: several patients presented visual and auditory hallucinations, agitation and nervousness, disorientation episodes, and behavioral alterations including aggressiveness. One of them presented seizures and another presented a global polyneuropathy. After switching the CNI from tacrolimus to cyclosporine, an improvement was observed in terms of clinical evolution in all of them.

When analyzing de novo development of cardiovascular risk factors and deterioration of renal function during follow-up after LTx, we came across the following results (Table 2).

**Table 2:** Development of de novo cardiovascular risk factors and renal impairment in the post-LTx period.

		CsA-group (n = 11)		Tac-group (n = 154)		p*
		LTRs susceptible to develop de novo events^a^	LTRs with de novo events	LTRs susceptible to develop de novo events^a^	LTRs with de novo events	
Cardiovascular risk factors	HTN	11	8 (72.73%)	122	41 (33.61%)	**0.003**
	Hyperlipidemia	10	6 (60.00%)	117	76 (64.96%)	0.739
	DM	11	0 (0%)	137	41 (29.93%)	0.067
Renal impairment		11	10 (90.91%)	152	115 (75.66%)	0.299

*HTN* arterial hypertension, *CsA-group* patients undergoing immunosuppressive treatment with cyclosporine as maintenance therapy, *DM* diabetes mellitus, *LTRs* lung transplant recipients, *Tac-group* patients undergoing immunosuppressive treatment with tacrolimus as maintenance therapy.

*The boldface type indicates differences that are statistically significant (*p* < 0.05).

^a^This column includes LTRs that, at the time of receiving their lung allograft, did not present each of the characteristics that were assessed in the study and were therefore the recipients that were susceptible to develop them.

There were 49 LTRs who developed HTN in the follow-up period (36.84%), with a higher percentage of this event within the CsA-group (72.73% vs. 33.61%; *p* = 0.003).

When reviewing new onset of hyperlipidemia, we found a total number of 82 LTRs (64.57%) who developed a de novo increase in cholesterol levels in their post-transplant blood tests, with no differences between both groups (60% in CsA-group vs. 64.96% in Tac-group; *p* = 0.739).

With regard to the analysis of the development of DM de novo, this event was only reported on LTRs undertaking tacrolimus treatment, where 41 out of 137 LTRs which could potentially develop this event, finally presented it (29.93%, *p* = 0.067).

When assessing the development of renal function impairment, we found 76.69% recipients who developed renal impairment from the total number of LTRs that were susceptible to this event. When looking at the type of immunosuppressant they were being treated with, 10 were documented in the group treated with cyclosporine (90.91% from the CsA-group) and 115 in the Tac-group (75.66% from this group) (*p* = 0.299).

The average drug doses were calculated for each of the timepoints evaluated. The results are shown in Table 3, along with the blood levels for each CNI drug.

**Table 3:** CNI blood levels and average doses at different post-LTx timepoints.

	Tac-group		CsA-group		
	Average blood level	Average dose (mg)	Average blood levels		Average dose (mg)
	C0 (mcg/L)		C0 (mcg/L)	C2 (mcg/L)	
3rd month	12.92 ± 3.21	7.75 (5.50–10.00)	247.07 ± 63.45	1030.88 ± 358.28	300.00 ± 50.00
6th month	12.42 ± 2.50	7.00 (5.00–9.13)	240.83 ± 45.10	1263.46 ± 356.02	250.00 ± 84.16
9th month	12.05 ± 2.33	6 (4.50–9.00)	249.30 ± 76.57	1218.95 ± 133.73	246.43 ± 41.90
1st year	11.27 ± 2.97	5.50 (4–8)	227.22 ± 53.53	1012.95 ± 123.31	275.00 ± 74.16
18 months	9.76 ± 2.48	5.00 (3.38–7.13)	205.42 ± 90.10	927.58 ± 143.47	200.00 (168.34–300.41)
2nd year	9.09 ± 2.21	4.00 (3.00–6.00)	167.57 ± 40.77	914.20 ± 231.38	225.00 ± 93.54
3rd year	8.53 ± 2.15	3.25 (2.13–5.00)	181.14 ± 83.24	860.50 ± 247.20	220.00 ± 75.83

C0: trough concentration. Blood level determination immediately before the next dose of a drug.

C2: blood level 2 h post-dose (in this case, 2 h post-dose of cyclosporine); CsA-group: patients undergoing immunosuppressive treatment with cyclosporine as maintenance therapy; Tac-group: patients undergoing immunosuppressive treatment with tacrolimus as maintenance therapy.

In the earliest stages LTRs showed higher blood drug levels (regardless the type of CNI), in good relationship with the higher doses employed in those stages.

In the CsA-group, we evaluated the relationship between the presence of ≥ 2 C0 levels above the proposed interval for each post-LTx stage and the occurrence of the different events studied, without finding any statistically significant relationship regarding the development of de novo HTN (*p* = 0.152, Fisher's exact test), hyperlipidemia (*p* = 0.545, Fisher's exact test) nor renal impairment (*p* = 1.000, Fisher's exact test).

In the Tac-group, we also evaluated the relationship between the presence of blood levels above the target for each post-LTx stage and the development of the different comorbidities. In this group of patients, due to their greater representation in number, the available number of tests was also higher. For each patient a record was made of whether they had ≥ 2, ≥ 3 or ≥ 4 tacrolimus trough levels above the upper limit of the target interval for each post-LTx stage. No statistically significant relationship was found for HTN, hyperlipidemia nor DM, but for those LTRs with ≥ 4 tacrolimus C0 determinations above the recommended blood levels we found a higher risk for developing renal impairment (OR 0.232, CI 0.087–0.623; *p* = 0.008).

Table 4 shows the data concerning the percentage of patients who developed each of the events in the post-LTx period, on the basis of the CNI prescribed, as calculated by Kaplan–Meier.

**Table 4:** Cumulative de novo cardiovascular risk factors and renal impairment in LTRs undertaking CNI treatment.

	CNI	Patients at risk to develop an event	Within the first 6 months post-LTx	Within 12 months	Within 18 months	Within 2 years	Within 3 years	Within 5 years	p*
HTN	Tac	122	18.0%	23.8%	28.7%	29.5%	31.1%	33.6%	**< 0.001**
	CsA	11	54.5%	54.5%	54.5%	63.6%	72.7%	72.7%	
Hyperlipi-demia	Tac	117	43.6%	51.3%	56.4%	59.8%	62.4%	64.1%	0.880
	CsA	10	40%	40%	40%	50%	60%	60%	
DM	Tac	137	16.8%	22.6%	24.8%	26.3%	29.2%	29.9%	0.065
	CsA	11	0%	0%	0%	0%	0%	0%	
Renal impairment	Tac	152	46.1%	62.5%	65.8%	71.1%	73.7%	75.7%	**0.047**
	CsA	11	63.6%	81.8%	90.9%	90.9%	90.9%	90.9%	

*HTN* arterial hypertension, *CNI* calcineurin inhibitor, *CsA* cyclosporine, *DM* diabetes mellitus, *Tac* tacrolimus.

*The boldface type indicates differences that are statistically significant (*p* < 0.05).

**Figure 1:**
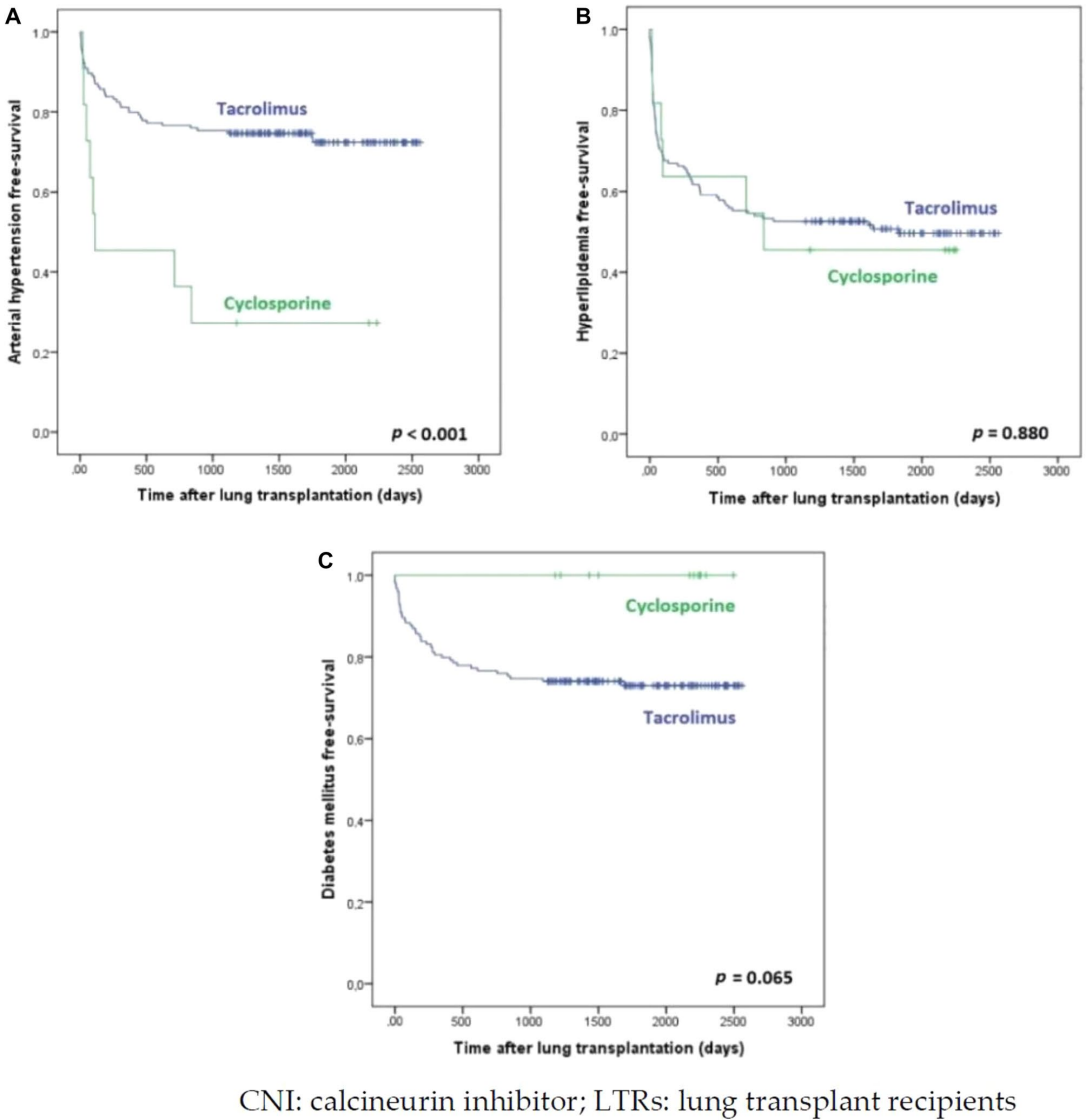
Development of cardiovascular risk factors according to the CNI drug prescribed in our cohort of LTRs in the post-transplantation period: (**A**) Development of arterial hypertension; (**B**) development of hyperlipidemia; (**C**) development of diabetes mellitus.

Figure 1 shows the Kaplan–Meier graphics obtained to HTN, hyperlipidemia and DM according to the CNI drug prescribed.

**Figure 2:**
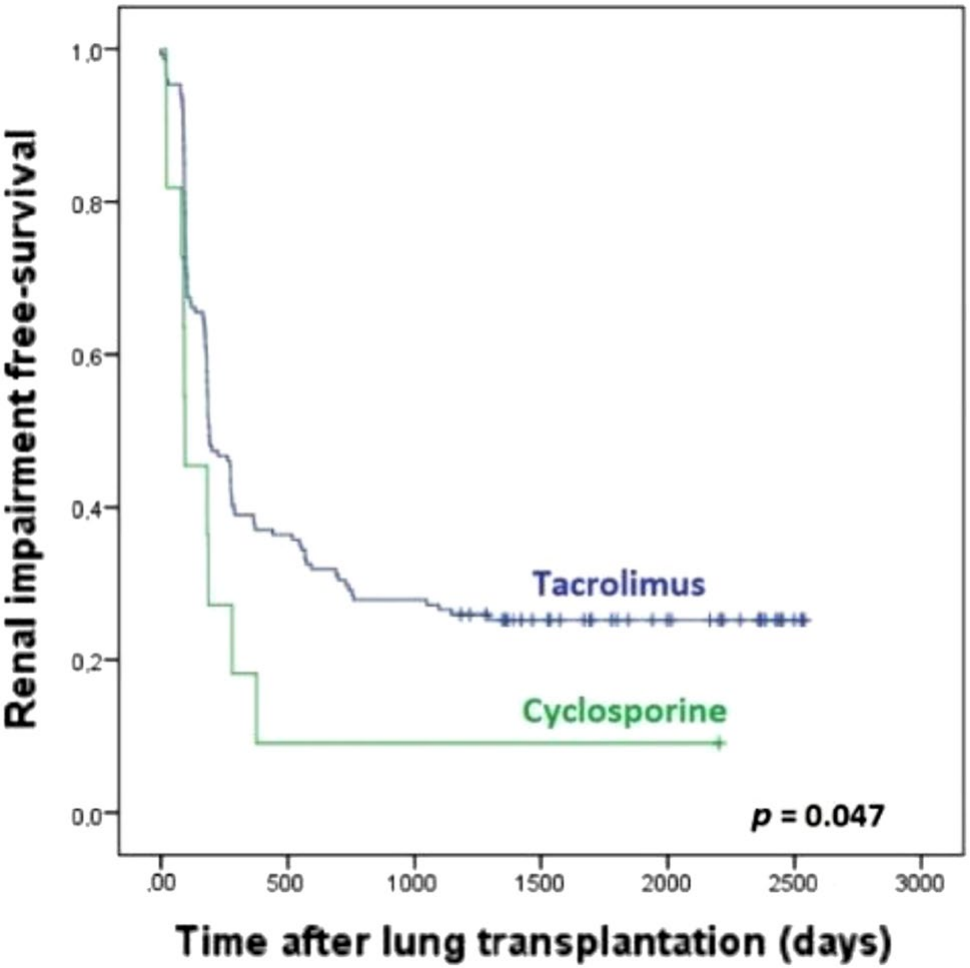
Development of renal impairment according to the CNI drug prescribed in our cohort of LTRs in the post-transplantation period.

With respect to the development of post-transplant renal function impairment, the nephrotoxicity rate was higher in the CsA-group (*p* = 0.047) (Fig. 2).

## Discussion

This study evaluates the development of cardiovascular risk factors and renal impairment related to CNI drugs in the post-transplant period in a group of LTRs.

In the last years, the ISHLT annual reports do no longer reflect this information for LTRs.

Our study aims to describe the situation in our population, which is representative from LTx at the North of Spain. To the best of our knowledge, this study is the first of our region to evaluate the association between the development of de novo cardiovascular risk factors and renal impairment in the post-transplant period of LTRs in our country.

In our work, a higher incidence of HTN was observed within the CsA-group. Although both tacrolimus and cyclosporine share the same mechanism of action, as both drugs belong to the CNI group, a lower incidence of cardiovascular effects has been reported with tacrolimus in previous studies^36^. This is due to the fact that they bind to different proteins (cyclosporine binds to cyclophilins, while tacrolimus binds to FK-binding proteins), so some differences in their effect can be expected. For example, in the case of tacrolimus, it has been reported that its effect on systemic vascular resistance is weaker than that reported with cyclosporine^37^.

Focusing on the specific effect of CNIs on the development of hyperlipidemia, a recent literature review comparing the effects of tacrolimus versus cyclosporine showed that there were a greater number of studies that associated the use of cyclosporine with an increased risk for hyperlipidemia (risk ratio 0.634; 95% CI 0.539–0.746, *p* < 0.001)^38^. For this reason, it is common in clinical practice to switch from cyclosporine to tacrolimus in an attempt to control cholesterol levels^39–41^.

In our cohort of LTRs, DM de novo was only documented in the Tac-group. The fact that this event was not observed in the CsA-group may be clearly influenced by the lower number of LTRs receiving this drug as CNI. Nevertheless, the incidence of DM de novo is known to be more frequent with the use of tacrolimus as a maintenance CNI according to medical literature. In this way, Webster et al. conducted a systematic review including publications on randomized controlled trials (RCTs) in renal transplantation. Data from 30 trials (n = 4102 patients) were included and, after a meta-analysis, it was noted that at one year, those recipients treated with tacrolimus had more insulin-requiring DM (risk ratio 1.86; CI 1.11–3.09)^36^. In a similar way, there is another meta-analysis of RCTs in kidney transplant from Liu et al.^42^, where eleven trials showed a significantly less risk for DM associated with cyclosporine treatment (risk ratio 1.38; 95% CI 1.06–1.79, *p* < 0.01). In the field of LTx, there is also a systematic review of RCTs that compares tacrolimus with cyclosporine^43^ where a higher rate of DM de novo was observed among LTRs undergoing tacrolimus treatment (OR 3.69; 95% CI 1.17–11.62; p = 0.03). In addition, it has also been described that the risk for DM increases with higher blood concentrations of tacrolimus^44^.

In our study, a higher incidence of impaired renal function was documented within the CsA-group. Although this finding, as the previous one, should also be considered with caution due to the same reason: a lower number of LTRs in our study receiving cyclosporine. When looking for data in the literature, CNI induced nephrotoxicity appears to be lower in patients undergoing tacrolimus treatment^45,46^.

With regard to the correlation between CNIs blood levels and the development of these events, some studies suggest that high levels of tacrolimus may be related to de novo appearance of DM or hyperlipidemia in the post-transplantation period^47^ and renal impairment has been described in association with increased blood levels of both CNIs^48^.

From a clinical point of view, it therefore becomes clear that it is important to have strategies to reduce, as far as possible, the potential development of toxicities related to the use of CNIs.

To specifically reduce CNIs-related nephrotoxicity, some strategies have been described. The medical administration of two or more immunosuppressants with different mechanisms of action is widely used nowadays, with the aim of reducing the prescribed dose of each drug and thereby minimizing the side effects of their administration^20,49^. A sample of this is what is sometimes referred to as “CNI minimization protocols”^50^. An example of the aforementioned consists in attempting to reduce CNIs blood levels, by the association to the immunosuppressive maintenance treatment of a drug from the mTOR inhibitors family. This type of association has proved that, besides achieving an improvement in renal function in patients with renal impairment, it does not increase the risk of acute rejection^51^. It has been described that the results are especially favorable when the deterioration of renal function is significant (glomerular filtration rate < 40 mL/min)^52^.

The addition of an mTOR inhibitor to the therapy makes it possible to reduce the CNI prescribed dose, maintaining good results in the evolution of the lung graft, and their results are the better the earlier this addition is made to the treatment^53^, but always being mindful that recovery from surgery should be awaited as mTOR inhibitors delay wound healing^54–56^. Although this strategy may be useful for patients whose main post-transplantation impact is on their renal function, this drug combination would not be a solution to the problem of the development of cardiovascular risk factors, as it has been documented that the concomitant use of steroids, CNI and mTOR inhibitors can lead to the development of HTN, hyperlipidemia and DM^57^.

It is therefore essential to adjust treatment on an individual basis, with the aim not only of improving graft survival, but also the impact on transplant recipients’ life quality (avoiding, as far as possible, the consequences of comorbidities derived from the toxicities of the drugs required after transplantation) and this must always be done on a case-by-case basis for each patient.

Among the limitations of this study, it should be firstly noted that it is a single center study with a retrospective basis, so information biases should be considered. The CsA-group was much smaller in number, so the observed results may not be representative. In our work, we have included a description of the two CNIs treatment options we have encountered, but due to differences in the number of patients with each type of CNI, we would like to point out that a statistically significant result in the CsA-group does not necessarily imply a correspondence in terms of clinical relevance.

When reviewing the available evidence in the medical literature, we found that there are several difficulties in exploring the toxicities associated with CNIs, not only because there is a lack of consensus on how to define each event, but also because different approaches are used for correlating events^58^.

Each of the events studied (HTN, hyperlipidemia, DM and impaired renal function) have multiple other conditioning factors that are beyond the scope of this study, but which should be taken into consideration.

Additionally, the available follow-up period is too short to assess cardiovascular risk factors' impact on the associated mortality risk, which also would certainly be of interest.

## Conclusions

In this review of 165 LTRs on CNI treatment, a higher incidence of developing HTN and deterioration of renal function was found within the CsA-group, while hyperlipidemia was observed with a slightly higher frequency in the Tac-group, that was not statistically significant. In the meanwhile, de novo appearance of DM was only described in patients treated with tacrolimus.

When assessing the relationship between the appearance of these events and CNIs blood levels, we found that LTRs with ≥ 4 tacrolimus C0 determinations above the upper limit of the proposed interval for each specific post-LTx stage had a higher risk for developing renal impairment. No other statistically significant association was found between supratherapeutic CNIs blood levels and the development of these comorbidities.

These events, widely described in other types of transplantation, are also confirmed in our LTRs sample, but further studies in this transplant population are required to confirm these data.

## Data Availability

All data generated or analyzed during this study are included in this article. Further enquiries can be directed to the corresponding author.

## Abbreviations

HTN: Arterial hypertension
C0: Trough concentration. Blood level determination immediately before the next dose of a drug
C2: Blood level 2 h post-dose of cyclosporine
CI: Confidence interval
CKD-EPI: Chronic Kidney Disease Epidemiology Collaboration
CNI: Calcineurin inhibitor
CsA-group: Patients undergoing immunosuppressive treatment with cyclosporine as maintenance therapy
DM: Diabetes mellitus
ISHLT: The International Society for Heart and Lung Transplantation
LTRs: Lung transplant recipients
LTx: Lung transplantation
mTOR inhibitors: Mammalian target of rapamycin inhibitors
NODAT: New onset diabetes mellitus after transplant
OR: Odds ratio
RCTs: Randomized controlled trials
SOT: Solid organ transplantation
Tac-group: Patients undergoing immunosuppressive treatment with tacrolimus as maintenance therapy
